# The Association between Serum Level of Vitamin D and Inflammatory Biomarkers in Hospitalized Adult Patients: A Cross-Sectional Study Based on Real-World Data

**DOI:** 10.1155/2024/8360538

**Published:** 2024-03-21

**Authors:** Xiaomin Zhang, Zhiqi Chen, Yi Xiang, Yiquan Zhou, Molian Tang, Jun Cai, Xinyi Xu, Hongyuan Cui, Yi Feng, Renying Xu

**Affiliations:** ^1^Department of Clinical Nutrition, Ren Ji Hospital, School of Medicine, Shanghai Jiao Tong University, Shanghai 200127, China; ^2^Department of Clinical Nutrition Center, Xin Hua Hospital, School of Medicine, Shanghai Jiao Tong University, Shanghai 200092, China; ^3^Department of Clinical Nutrition, Long Hua Hospital affiliated to Shanghai University of Traditional Chinese Medicine, Shanghai 200031, China; ^4^University of Michigan, LSA 500 S, State Street, Ann Arbor, MI 48109, USA; ^5^Department of General Surgery, Beijing Hospital, National Center of Gerontology, Institute of Geriatric Medicine, Chinese Academy of Medical Science, Beijing 100730, China; ^6^Department of Nutrition, College of Health Science and Technology, Shanghai Jiao Tong University School of Medicine, Shanghai 200025, China

## Abstract

**Objective:**

The association between vitamin D status and inflammation remains unclear in hospitalized patients.

**Materials and Methods:**

We performed the current study based on real-world data from two teaching hospitals. Serum level of vitamin D (assessed by 25-hydroxyvitamin D) was evaluated within 2 days after admission. All the patients were further classified into three groups: deficiency (<12 ng/mL), insufficiency (12–20 ng/mL), and adequate (≥20 ng/mL). White blood cell (WBC) count, serum level of C-reactive protein (CRP), and procalcitonin were also measured and used to evaluate inflammation. Other potential covariates were abstracted from medical records. Charlson comorbidity index (CCI) was calculated to assess the severity of disease.

**Results:**

A total number of 35,528 hospitalized adult patients (21,171 men and 14,357 women) were included. The average age and BMI were 57.5 ± 16.2 years and 23.4 ± 3.7 kg/m^2^, respectively, while medium vitamin D level was 16.1 ng/mL (interquartile range: 11.4 ng/mL, 21.6 ng/mL) and median CCI was one point (interquartile range: 0 point, two points). The prevalence of deficiency and insufficiency was 28.0% and 40.5%. Multivariate linear regression model showed that serum level of vitamin D was significantly associated with WBC and CRP but not associated with procalcitonin. Each standard deviation (≈7.4 ng/mL) increase in vitamin D was associated with a decrease in WBC by 0.13 × 10^9^/mL (95% CI: 0.2 × 10^9^/mL, 0.06 × 10^9^/mL) and 0.62 mg/L (95% CI: 0.88 mg/L, 0.37 mg/L) for CRP. Subgroup analysis and sensitivity analysis (excluding those whose eGFR <60 ml/min/1.73 m^2^, those whose daily calorie intake <1,000 kcal, and those who were recruited from Xin Hua hospital) generated similar results.

**Conclusions:**

The deficiency and insufficiency of vitamin D in the hospitalized adult patients was very common. However, the results should be interpreted with caution for limited representation of the whole inpatients. Low level of vitamin D was associated with inflammatory biomarkers, which provide the evidences to early intervention for lower the risk of infection.

## 1. Introduction

Since vitamin D was first discovered by McCollum et al. [1], many efforts have been put into elucidating the role of vitamin D in maintaining human health. Besides its main role in calcium metabolism and bone health, vitamin D is believed to be involved in immune regulation [2], development of type 2 diabetes [3], and cardiovascular disease [4], cell differentiation [5], and even reproduction [6].

The deficiency and insufficiency of vitamin D are very common in community population. The 2014 National Health and Nutrition Examination Survey found that 5% of the population were confirmed with deficiency (<12 ng/mL) and 18% of whom were with insufficiency (12–19 ng/mL) [7]. Although endogenous production of vitamin D is mainly produced by human skin after sunlight exposure and thus individuals lived in high latitude areas are in high risk of vitamin D deficiency, persons lived in Abu Dhabi Emirates [8] and Bangladesh [9] (the area with enough sun exposure) were also confirmed with high prevalence of vitamin D deficiency.

The prevalence of vitamin D was reported in hospitalized patients with inflammatory bowel diseases [10], autoimmune diseases [11], and cancer [12]; however, limited studies were performed on patients from general wards. A retrospective study including 8,861 Swiss patients reported a high prevalence of vitamin D deficiency: 51% of population were vitamin D deficient with levels <20 ng/mL, including 1,860 (21.0%) with levels <10 ng/mL [13]. Another small sample size (*n* = 384) study reported that the prevalence of vitamin D was 73.6% based on the criteria of less than 30 ng/mL [14]. Further, the association between vitamin D and inflammation was controversary. A cross-sectional study reported that vitamin D was associated with surgical site [15] and bloodstream infection [16] while not associated with hepatic inflammation [17] in patients with chronic hepatitis C and not associated with high-sensitivity C-reactive protein (CRP) in patients with HIV [18]. Another meta-analysis including observational studies reported that participants with vitamin D deficiency were in high risk of developing sepsis compared to controls [19]. Using procalcitonin as an inflammatory biomarker also generated inconsistent results [20–22].

Therefore, we performed the current retrospective studies based on real-world data to evaluate the association between vitamin D and inflammation, which was evaluated by three biomarkers (CRP, white blood cell (WBC), and procalcitonin).

## 2. Materials and Methods

**Figure 1:**
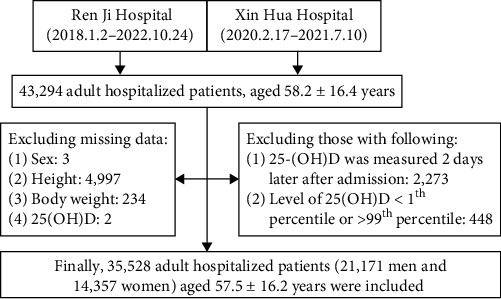
The process of sample recruitment.

This study is a retrospective, cross-sectional study based on real-world data. All the potential participants were recruited from Ren Ji (January 1, 2018 to October 31, 2022) and Xin Hua Hospital (January 1, 2020 to December 31, 2021). Participants recruited from Ren Ji Hospital were younger, with lower proportion of women, higher Charlson Comorbidity Index (CCI), higher procalcitonin, lower CRP, lower number of WBC, and lower level of vitamin D, compared with those from Xin Hua Hospital (Table S1). We performed a sequential process of sample recruitment. First, we excluded those with missing data on sex (*n* = 3), height (*n* = 4,997), body weight (*n* = 234), and 25(OH)D (*n* = 2). Then, we excluded those whose serum level of vitamin D was measured 2 days after admission (*n* = 2,273), and those extreme values (serum level of vitamin D < 1^th^ percentile or >99^th^ percentile, *n* = 448). Finally, 35,528 adult hospitalized patients (21,171 men and 14,357 women) aged 57.5 ± 16.2 years were included (details are shown in Figure 1). The study protocol was approved by the Ethical Committee of Ren Ji (No. LY-2022-057-B) and Xin Hua Hospital (No. XHEC-C-2023-014-1). As a retrospective study, patients' written consent was waived by the same Ethical Committee mentioned above.

### 2.1. Clinical Information

Clinical information was abstracted from medical records. CCI was used to assess the severity of diseases [23]. In brief, height and body weight were measured by trained nurses at hospital admission. Venous blood samples were drawn within 2 days after hospital admission and transfused into vacuum tubes containing EDTA in the morning after patients were fasted for at least 8 hr. Fasting blood glucose (FBG), albumin, total cholesterol (TC), total triglycerides (TG), alanine transferase (ALT), aspartate transaminase (AST), alkaline phosphatase (AKP), gamma glutamyl-transferase (GGT), total bilirubin (TBIL), direct bilirubin (DBIL) were measured by enzyme-linked immunosorbent method. Estimated glomerular filtration rate (eGFR-EPI) was calculated by the Chronic Kidney Disease Epidemiology Collaboration (CKD-EPI) equation [24]. All the blood samples were analyzed in the Clinical Laboratory Center of Ren Ji and Xin Hua Hospital. The same instruments and methods were used in both hospitals to assess biochemical parameters. Demographical information (age and sex), primary disease for hospital admission, history of chronic disease, and admission date were also abstracted. Dietary intake was assessed based on medical orders. If it differed between the 2 days after the admission, the last one was used.

The abnormal of liver enzymes were defined if one of the following was greater than twofold of upper limit based on laboratory recommendations: ALT ≥ 120, AST ≥ 150, AKP ≥ 250, and GGT ≥ 100 U/L [25]. Cholestasis was also determined if TBIL (≥34.2 *μ*mol/L) or DBIL (≥13.6 *μ*mol/L) was greater than twofold of upper limit [25]. Dyslipidemia was diagnosed if TC ≥ 5.72 mmol/L or TG ≥ 1.7 mmol/L [26].

### 2.2. Exposure and Outcome

In our hospital, we recommended those with inadequate sun exposure (e.g., systemic lupus erythematosus), limited oral intake, impaired intestinal absorption, or with clinical signs of vitamin D deficiency, to measure serum level of vitamin D. Serum level of vitamin D was the exposure in the current study. We assessed serum level of vitamin D by measuring serum level of levels of 25-hydroxyvitamin D (1 nmol/L = 2.5 *μ*g/L = 2.5 ng/mL) through an electrochemiluminescence immunoassay. The intra- and inter-CV were 2.2% and 3.4%, respectively. The lower limit of detection was 3.0 ng/mL while it is 70.0 ng/mL for the high limit of detection. All the patients were classified into three groups based on serum level of 25(OH)D as follows: deficiency (<12.0 ng/mL), insufficiency (12.0–20.0 ng/mL), and adequate (≥20.0 ng/mL) [27]. We used three inflammatory biomarkers, including WBC, CRP, and procalcitonin, to assess inflammatory status. Usually, WBC and CRP were biomarkers for systemic inflammation while procalcitonin was the one for bacterial infection [28, 29]. The three inflammatory biomarkers were treated as outcomes. WBC was analyzed by an automatic hematology analyzer (XN-10, Sysmex, Japan). Serum level of CRP was measured by immunoturbidimetric method (PA990 analyzer, Lifotronic Technology, Shenzhen, China). Serum level of procalcitonin was measured by electrochemical luminescence method (Cobas e411 analyzer, Roche Diagnostics GmbH, Mannheim, Germany). The lower limit of detection was 0.5 mg/L for CRP and 0.01 mg/L for procalcitonin measurement.

### 2.3. Statistical Analysis

In the descriptive statistics, continuous variables were presented as average and standard deviations (SDs), if it is in normal distribution, while medium and interquartile ranges were in abnormal distribution. Categorical variables were shown as proportion.

We used the general linear model analysis to evaluate the association between serum vitamin D and three inflammatory biomarkers (WBC, CRP, and procalcitonin), group differences were tested by multiple ANOVA. We adjusted potential covariates in different models: model 1, adjusting for age (years) and sex (men vs. women); model 2, adjusting for sex (men vs. women), age (year), hospital (Ren Ji vs. Xin Hua), season (spring, summer, autumn, or winter), BMI (<18.5, 18.5–23.9, or ≥24.0 kg/m^2^), anemia (yes vs. no), albumin (<35.0 vs. ≥35.0 g/L), FBG (<6.0 vs. ≥6.0 mmol/L), serum level of liver enzymes (normal vs. abnormal), cholestasis (yes vs. no), eGFR-EPI (<60.0 vs. ≥60.0 mL/min/1.73 m^2^), dyslipidemia (yes vs. no), CCI (0, 1–2, or ≥3 points), and dietary intake (<500, 500–1,000, or ≥1,000 kcal/day).

Likelihood-ratio tests were conducted to examine statistical interactions between sex, and age, in relation to the conversion by comparing −2 log likelihood *χ*^2^ between nested models with and without the cross-product variables. Subgroup analyses were performed regardless of the statistical significance of interaction. We further performed three sensitivity analyses (excluding those whose eGFR < 60 mL/min/1.73 m^2^ [30], whose daily calorie intake <1,000 kcal [31], and who were recruited from Xin Hua Hospital) to test the robustness of main analysis. A *P*-values < 0.05 was considered significant, and the analysis of the data was carried out using SAS software (SAS Institute, Inc., Cary, NC, USA).

## 3. Results

A total number of 35,528 adult hospitalized patients (21,171 men and 14,357 women) aged 57.5 ± 16.2 years were included in the study. The average age and BMI and medium CCI were 57.5 ± 16.2 years, 23.4 ± 3.7 kg/m^2^, one point (interquartile range: 0 point, two points), respectively. The main reason for hospital admission was gastrointestinal diseases (55.2%), then followed by neurological diseases (16.6%), hematological diseases (10.1%), and malnutrition and electrocyte disorders (7.2%) (Table S2). Serum level of vitamin D was associated with all the clinical parameters except for daily dietary intake (Table 1).

The medium vitamin D level was 16.1 ng/mL (interquartile range: 11.4, 21.6 ng/mL). Serum level of vitamin D was obviously higher in patients admitted in summer and autumn than those who were admitted in spring and winter (Table 2). Interestingly, age, BMI, FBG, liver and renal function, and lipid profile differed obviously among different seasons (Table 2).

The prevalence of deficiency and insufficiency was 28.0% (*n* = 9,955) and 40.5% (*n* = 14,394) in the current study population. Multivariate linear regression model showed that serum level of vitamin D was significantly associated with WBC and CRP, in fully adjusted model. Serum level of vitamin D was associated with procalcitonin after adjustment of sex and age; however, the association lost significance in further adjustment of other covariates. Each standard deviation (≈7.4 ng/mL) increase in vitamin D was associated with a decrease in WBC by 0.13 × 10^9^/mL (95% CI: 0.2 × 10^9^/mL, 0.06 × 10^9^/mL) and 0.62 mg/L (95% CI: 0.88, 0.37 mg/L) for CRP (Table 3). Generally, subgroup analysis (stratified by sex and age) and sensitivity analysis (excluding those whose eGFR <60 mL/min/1.73 m^2^, whose daily calorie intake <1,000 kcal, and who were recruited from Xin Hua Hospital) generated similar results (Tables S3 and S4).

## 4. Discussion

In the current retrospective study based on real-world data, we found that the proportion was 65.8% for those participants whose serum level of vitamin D was less than 20 ng/mL. The results could provide some evidences to the situation of vitamin D insufficiency or deficiency, but over-representation needs to be avoided because the sample size (*n* = 35,528) only represented a very small proportion of the whole hospital patients (about 7% during the recruitment period). Further, vitamin D was associated with CRP and WBC, but not procalcitonin, after adjustment of age, sex, season, hospital, CCI, and other covariates.

A retrospective study including 8,861 Swiss patients reported a high prevalence of vitamin D deficiency: 51% of population were vitamin D deficient with levels <20 ng/mL, including 1,860 (21.0%) with levels <10 ng/mL [13]. Another small sample size (*n* = 384) study reported that the prevalence of vitamin D was 73.6% based on the criteria of less than 30 ng/mL [14]. Together with our study, the estimated prevalence of vitamin D deficiency and insufficiency was over half of hospitalized patients, regardless of the differences in criteria, study population, and ethnicity.

Numerous studies have reported a high prevalence of vitamin D deficiency in patients with COVID-19 and its association with clinical outcomes [32, 33]; however, whether vitamin D was associated with inflammation was still unclear. We found that serum level of vitamin D was associated with two inflammatory biomarkers: with the increase in serum level of vitamin D, the significant decrease in WBC and CRP was confirmed even after adjustment of age, sex, season, and severity of disease (assessed by CCI). A retrospective study including 23,603 adult patients whose serum level of vitamin D was measured before admission reported patients whose serum level of vitamin D was less than 15 ng/mL were in high risk of blood infection (adjusted OR = 1.29, 95% CI: 1.06, 1.57) [16]. However, about 51.8% (12,224/23,603) of vitamin D measurement was performed 90 days or more before admission [16]. Another case-control study reported that patients whose serum level of vitamin D was less than 10 ng/mL were associated with IL-6 (interleukin-6) by 4.6% but not associated with high-sensitivity CRP [18]. In a single-center retrospective study including 655 critically care patients, although mortality differed significantly between patients with and without vitamin D deficiency and insufficiency, serum vitamin D level was not associated with CRP [20]. The possible explanation was that high-sensitivity CRP might be a good indicator for chronic diseases, but not suitable for acute disease. Another meta-analysis reported that patients with vitamin D deficiency were at high risk of developing sepsis compared to controls [19]. The consistent results could be confirmed in observational studies, but not in RCT data [34]. The mechanism of anti-inflammation of vitamin D could be explained by the inhibition the translocation of NF*κ*B to nucleus, thus reducing the production of proinflammatory cytokines [2]. We did not find the association between vitamin D and procalcitonin, which was also supported by other studies [20, 21]. Another observational study reported that vitamin D level at admission was associated with procalcitonin; however, the interval time between vitamin D measurement and procalcitonin measurement was not reported; further, the association was analyzed by simple correlation and did not take potential covariates into consideration [22].

Sex, age, season (summer and autumn vs. spring and winter), severity of disease, and liver and renal function contributed to the deficiency of vitamin D (data were not shown). Further, the association between vitamin D and inflammation seemed stronger in men and in those aged 65 years or less, compared to their counterparts.

The strength of the current study included a larger sample size based on real-world data and deliberately taking a series of potential covariates (e.g., season and severity of disease) into consideration. However, there were also some limitations. First, we did not know the change in vitamin D and whether this change could lead to a decrease in inflammatory biomarkers. Further, we also did not know if the change in inflammation could result significant improvement in clinical outcomes such as hospital duration and mortality was not evaluated. Second, we used 25(OH)D, but not the active form of vitamin D (cholecalciferol), to evaluated vitamin D status. However, because half-life time of cholecalciferol was short (4–15 hr) and instrument used to evaluate cholecalciferol was expensive, 25(OH)D was widely used [7, 16, 35]. Third, we did not collect the information on vitamin D supplements, which might interaction with the association between vitamin D and inflammation. Finally, as a retrospective study, causal association could not be generated. Although many observational studies reported the association between vitamin D and inflammation; however, supplement of vitamin D did not generate consistent results in community population [36] and hospitalized patients [37, 38]. Further, vitamin D was assessed in those with high risk of vitamin D deficiency, thus we could not exclude the possible selection bias and the generalizability was limited. As a retrospective study, information on the use of corticosteroids was deficient, which might have effects on both serum level of vitamin D and inflammatory biomarkers [39, 40]. A well-designed prospective study with these information is needed to duplicate our results.

## 5. Conclusions

The prevalence of vitamin D deficiency was over 50% in hospitalized patients. Further, serum level of vitamin D was associated with inflammation. Our results support the recommendation that measurement of vitamin D in those with high risk (such as systemic lupus erythematosus and inflammatory bowel disease) might be helpful to early diagnosis and intervention, thus reducing the risk of infection.

## Figures and Tables

**Table 1:** Basic characteristics across different vitamin D groups in 35,528 Chinese adult hospitalized patients.

Parameter	Group	Serum level of 25(OH)D (ng/mL)			*P* value
		<12 (*n* = 9,955)	12–20 (*n* = 14,394)	≥20 (*n* = 11,179)	
Age (year)	N/A	56.2 ± 17.6	57.2 ± 16.0	59.0 ± 15.2	<0.001
Sex, *n* (%)	Men	5,339 (53.6)	8,382 (58.2)	7,450 (66.6)	<0.001
	Women	4,616 (46.4)	6,012 (41.8)	3,729 (33.4)	—
Hospital *n* (%)	Ren Ji	9,205 (92.5)	13,001 (90.3)	10,172 (91.0)	<0.001
	Xin Hua	750 (7.5)	1,393 (9.7)	1,007 (9.0)	—
Season *n* (%)	Spring	2,976 (29.9)	3,322 (23.1)	1,818 (16.3)	<0.001
	Summer	2,417 (24.3)	4,504 (31.3)	3,886 (34.8)	—
	Autumn	2,295 (23.1)	4,309 (29.9)	4,281 (38.3)	—
	Winter	2,267 (22.8)	2,259 (15.7)	1,194 (10.7)	—
BMI (kg/m^2^)	N/A	24.0 ± 4.0	23.4 ± 3.6	22.9 ± 3.3	<0.001
FBG (mmol/L)	N/A	5.9 ± 2.5	6.0 ± 2.4	5.9 ± 2.3	0.003
TC (mmol/L)	N/A	4.7 ± 1.8	4.5 ± 1.2	4.4 ± 1.1	<0.001
TG (mmol/L)	N/A	1.39 (0.97, 2.07)	1.37 (0.97, 1.98)	1.37 (0.97, 1.97)	0.02
Albumin (g/L)	N/A	36.9 ± 7.3	40.8 ± 4.9	41.9 ± 4.5	<0.001
Prealbumin (mg/L)	N/A	222.6 ± 73.8	238.1 ± 62.3	247.6 ± 59.7	<0.001
ALT (U/L)	N/A	16 (11, 25)	17 (12, 26)	18 (13, 26)	<0.001
AST (U/L)	N/A	19 (14, 25.6)	19 (15, 25)	19 (16, 25)	<0.001
AKP (U/L)	N/A	75 (60, 93)	74 (61, 90)	74 (61, 90)	0.001
GGT (IU/L)	N/A	22 (15, 27)	22 (15, 36)	23 (16, 36)	0.002
TBIL (mmol/L)	N/A	8.5 (5.9, 12.2)	9.7 (7.1, 13.2)	10.1 (7.5, 13.7)	<0.001
DBIL (mmol/L)	N/A	2.7 (1.8, 4.0)	3.1 (2.2, 4.2)	3.2 (2.3, 4.4)	<0.001
eGFR-EPI (mL/min/1.73 m^2^)	N/A	92.7 (55.1, 110.6)	97.8 (79.2, 111.5)	96.4 (78.5, 109.4)	<0.001
CRP (mg/L)	N/A	1.3 (0.5, 6.6)	1.0 (0.5, 3.3)	0.8 (0.5, 2.6)	<0.001
WBC (10^9^/L)	N/A	6.2 (5.0, 7.8)	6.0 (4.9, 7.4)	6.0 (4.9, 7.2)	<0.001
Procalcitonin (ng/mL)	N/A	2.2 (1.7, 2.7)	2.2 (1.5, 2.7)	2.2 (1.6, 2.7)	<0.001
CCI (point)	N/A	1.0 (0.0, 2.0)	1.0 (0.0, 2.0)	0.0 (0.0, 2.0)	<0.001
Diet intake, *n* (%)	<500 kcal/day	448 (4.5)	660 (4.6)	521 (4.7)	0.8
	500–1,000 kcal/day	383 (3.9)	513 (3.6)	399 (3.6)	—
	≥1,000 kcal/day	9,124 (91.7)	13,321 (91.9)	10,259 (91.8)	—

*Notes*: BMI, body mass index; FBG, fasting blood glucose; TC, total cholesterol; TG, triglycerides; ALT, alanine transferase; AST, aspartate transaminase; AKP, alkaline phosphatase; GGT, gamma glutamyl-transferase; TBIL, total bilirubin; DBIL, direct bilirubin; eGFR-EPI, estimated glomerular filtration rate calculated by Chronic Kidney Disease Epidemiology Collaboration equation; CRP, C reactive protein; WBC, white blood cell count; CCI, Charlson comorbidity index. CCI was determined without information on HIV infection or AIDS. If continuous data were in abnormal distribution, data were shown as median and quartile range. Categorical data were shown as numbers and proportions.

**Table 2:** Basic characteristics across seasons in 35,528 Chinese adult hospitalized patients.

Parameter	Group	Season				*P* value
		Spring	Summer	Autumn	Winter	
Age (year)	N/A	58.3 ± 16.0	56.4 ± 16.5	57.6 ± 16.2	58.4 ± 16.0	<0.001
Sex, *n* (%)	Men	4,800 (59.1)	6,474 (59.9)	6,483 (59.6)	3,414 (59.7)	0.7
	Women	3,316 (40.9)	4,333 (40.1)	4,402 (40.4)	2,306 (40.3)	—
Hospital, *n* (%)	Ren Ji	7,492 (92.3)	10,382 (96.1)	9,564 (87.9)	4,940 (86.4)	<0.001
	Xin Hua	624 (7.7)	425 (3.9)	1,321 (12.1)	780 (13.6)	—
BMI (kg/m^2^)	N/A	25.1 ± 2.6	23.2 ± 2.5	20.9 ± 2.9	26.4 ± 4.6	<0.001
FBG (mmol/L)	N/A	6.0 ± 2.3	5.8 ± 2.2	6.0 ± 2.5	6.2 ± 2.5	<0.001
TC (mmol/L)	N/A	4.6 ± 1.4	4.5 ± 1.4	4.5 ± 1.3	4.6 ± 1.4	<0.001
TG (mmol/L)	N/A	1.36 (0.96, 1.98)	1.4 (0.99, 2.07)	1.38 (0.97, 2.0)	1.35 (0.95, 1.94)	<0.001
Albumin (g/L)	N/A	39.6 ± 6.0	40.1 ± 6.0	40.4 ± 5.8	39.9 ± 5.9	<0.001
Prealbumin (mg/L)	N/A	236.7 ± 68.5	239.7 ± 66.0	235.7 ± 64.1	232.9 ± 63.8	<0.001
ALT (U/L)	N/A	17 (12, 25)	16 (11, 25)	17 (12, 26)	17 (12, 26)	<0.001
AST (U/L)	N/A	18 (15, 24)	18 (15, 25)	19 (15, 26)	19 (16, 26)	<0.001
AKP (U/L)	N/A	74 (61, 91)	74 (61, 91)	74 (61, 90)	74 (61, 91)	0.45
GGT (IU/L)	N/A	22 (15, 35)	22 (15, 36)	23 (16, 37)	23 (15, 37)	<0.001
TBIL (mmol/L)	N/A	9.4 (6.8, 12.9)	9.4 (6.8, 13.1)	9.8 (7.1, 13.5)	9.4 (6.8, 12.9)	<0.001
DBIL (mmol/L)	N/A	3.0 (2.1, 4.2)	3.1 (2.2, 4.3)	3.1 (2.1, 4.3)	2.8 (2.0, 3.9)	<0.001
eGFR-EPI (mL/min/1.73 m^2^)	N/A	95.7 (74.4, 109.6)	96.7 (72.8, 111.7)	96.2 (74.2, 110.3)	96.2 (75.9, 109.9)	0.07
CRP (mg/L)	N/A	0.9 (0.5, 3.4)	0.9 (0.5, 3.7)	1.0 (0.5, 3.9)	1.0 (0.5, 3.6)	<0.001
WBC (10^9^/L)	N/A	6.0 (4.9, 7.4)	6.0 (4.9, 7.3)	6.1 (5.0, 7.5)	6.0 (4.9, 7.4)	<0.001
Procalcitonin (ng/mL)	N/A	2.2 (1.6, 2.7)	2.2 (1.8, 2.7)	2.2 (1.5, 3.4)	2.1 (1.4, 2.6)	<0.001
25(OH)D (ng/mL)	N/A	14.2 (10.0, 19.4)	17.3 (12.5, 22.5)	17.8 (12.9, 23.2)	13.7 (9.7, 18.9)	<0.001
CCI (point)	N/A	1 (0, 2)	1 (0, 2)	1 (0, 2)	1 (0, 2)	<0.001
Diet intake, *n* (%)	<500 kcal/day	423 (5.3)	511 (4.7)	408 (3.8)	283 (5.0)	0.03
	500–1,000 kcal/day	273 (3.4)	371 (3.4)	432 (4.0)	219 (3.8)	—
	≥1,000 kcal/day	7,416 (91.4)	9,925 (91.8)	10,045 (92.3)	5,218 (91.2)	—

*Notes*: BMI, body mass index; FBG, fasting blood glucose; TC, total cholesterol; TG, triglycerides; ALT, alanine transferase; AST, aspartate transaminase; AKP, alkaline phosphatase; GGT, gamma glutamyl-transferase; TBIL, total bilirubin; DBIL, direct bilirubin; eGFR-EPI, estimated glomerular filtration rate calculated by Chronic Kidney disease epidemiology collaboration equation; CRP, C reactive protein; WBC, white blood cell count; CCI, Charlson comorbidity index. CCI was determined without information on HIV infection or AIDS. If continuous data were in abnormal distribution, data were shown as median and quartile range. Categorical data were shown as numbers and proportions.

**Table 3:** The association between vitamin D and inflammatory biomarkers in 35,528 Chinese adult hospitalized patients.

Inflammatory biomarkers	Serum level of 25(OH)D (ng/mL)			Each SD increment (≈7.4 ng/mL)	*P*_trend_
	<12 (*n* = 9,955)	12–20 (*n* = 14,394)	≥20 (*n* = 11,179)		
WBC					
Age- and sex-adjusted model	0.59 (0.42, 0.76)	0.09 (−0.07, 0.25)	Ref (0)	−0.24 (−0.31, −0.18)	<0.001
Multivariate model	0.31 (0.13, 0.49)	0.03 (−0.12, 0.19)	Ref (0)	−0.13 (−0.20, −0.06)	<0.001
CRP					
Age- and sex-adjusted model	6.68 (6.02, 7.33)	2.22 (1.62, 2.82)	Ref (0)	−2.6 (−2.85, −2.35)	<0.001
Multivariate model	1.59 (0.93, 2.26)	1.06 (0.49, 1.63)	Ref (0)	−0.62 (−0.88, −0.37)	<0.001
Procalcitonin					
Age- and sex-adjusted model	0.03 (−0.001, 0.06)	−0.04 (−0.08, −0.02)	Ref (0)	−0.02 (−0.03, −0.005)	0.006
Multivariate model	0.007 (−0.02, 0.04)	−0.02 (−0.05, 0.002)	Ref (0)	0.0002 (−0.01, 0.01)	0.97

*Notes*: Multivariate model was adjusted by sex (men vs. women), age (year), hospital (Ren Ji vs. Xin Hua), season (spring, summer, autumn, or winter), BMI (<18.5 kg/m^2^, 18.5–23.9 kg/m^2^, or ≥24.0 kg/m^2^), anemia (yes vs. no), albumin (<35.0 g/L vs. ≥35.0 g/L), fasting blood glucose (<6.0 mmol/L vs. ≥6.0 mmol/L), serum level of liver enzymes (normal vs. abnormal), cholestasis (yes vs. no), eGFR-EPI (<60.0 vs. ≥60.0 mL/min/1.73 m^2^), dyslipidemia (yes vs. no), CCI (0, 1–2, or ≥3 points), diet intake (<500 kcal/day, 500–1,000 kcal/day, or ≥1,000 kcal/day). Anemia was confirmed as following: serum hemoglobin <120 g/L in men or <110 g/L in women. Abnormal liver enzyme was confirmed if any of the following: alanine transferase ≥ 120 U/L, or aspartate transaminase ≥ 150 U/L, or alkaline phosphatase ≥ 250 U/L, or gamma glutamyl-transferase ≥ 100 IU/L. Cholestasis was confirmed if either of the following: total bilirubin ≥ 34.2 *μ*mol/L or direct bilirubin ≥ 13.6 *μ*mol/L. Dyslipidemia was confirmed if either of the following: total cholesterol ≥ 5.72 mmol/L or total triglycerides ≥1.7 mmol/L.

## Data Availability

The data and SAS code are available upon reasonable request from the corresponding author (Renying Xu: 721001735@shsmu.edu.cn).

## Supplementary Materials

Supplementary Materials The comparison between different hospitals, the primary disease for hospital admission, and the results for subgroup and sensitivity analysis, were shown in Supplementary files.
