# Supplementary material for: The Association between Serum Level of Vitamin D and Inflammatory Biomarkers in Hospitalized Adult Patients: A Cross-Sectional Study Based on Real-World Data
Source: Mediators Inflamm. 2024 Mar 21;2024:8360538. doi: 10.1155/2024/8360538 (PMC10978080; doi:10.1155/2024/8360538)
Supplement: Supplementary Materials — The comparison between different hospitals, the primary disease for hospital admission, and the results for subgroup and sensitivity analysis, were shown in Supplementary files. [file 8360538.f1.docx]

**Supplemental Table 1.** The comparison of clinical characteristics between Ren Ji and Xin Hua Hospital

| Parameter | Group | Ren Ji Hospital  (n=32,378) | Xin Hua Hospital  (n=3,150) | P value |
| --- | --- | --- | --- | --- |
| Age, year | N/A | 56.9±16.3 | 63.3±14.9 | <0.001 |
| Sex, n (%) | Men, | 19,552 (60.4) | 1,619 (51.4) | <0.001 |
|  | Women | 12,826 (39.6) | 1,531 (48.6) |  |
| BMI, kg/m^2^ | N/A | 23.4±3.7 | 23.5±3.6 | 0.29 |
| FBG, mmol/L | N/A | 5.8±2.2 | 7.2±3.4 | <0.001 |
| TC, mmol/L | N/A | 4.5±1.4 | 4.3±1.1 | <0.001 |
| TG, mmol/L | N/A | 1.38 (0.98, 2.02) | 1.25 (0.89, 1.81) | <0.001 |
| Albumin, g/L | N/A | 40.1±6.1 | 39.3±4.3 | <0.001 |
| Pre-albumin, mg/L | N/A | 240.0±65.6 | 201.2±55.8 | <0.001 |
| ALT, U/L | N/A | 17 (12, 25) | 18 (13, 27) | <0.001 |
| AST, U/L | N/A | 19 (15, 24) | 22 (17, 32) | <0.001 |
| AKP, U/L | N/A | 74 (61, 90) | 77 (63, 97) | <0.001 |
| GGT, IU/L | N/A | 22 (15, 36) | 23 (16, 41) | <0.001 |
| TBIL, mmol/L | N/A | 9.4 (6.8, 12.9) | 11.1 (8.3, 15.4) | <0.001 |
| DBIL, mmol/L | N/A | 3.1 (2.2, 4.3) | 2.5 (0.6, 3.9) | <0.001 |
| eGFR-EPI, ml/min/1.73m^2^ | N/A | 96.1 (72.7, 110.6) | 98.0 (84.6, 109.9) | <0.001 |
| CRP, mg/L | N/A | 0.8 (0.5, 3.3) | 2.0 (1.0, 7.0) | <0.001 |
| WBC, 10^9^/L | N/A | 6.0 (4.9, 7.4) | 6.3 (5.0, 8.0) | <0.001 |
| Procalcitonin, ng/ml | N/A | 2.3 (1.8, 2.8) | 0.2 (0.17, 0.24) | <0.001 |
| CCI, point | N/A | 1 (0, 2) | 0 (0, 1) | <0.001 |
| 25-(OH)-D, ng/ml | N/A | 16.1 (11.3, 21.6) | 16.6 (12.2, 21.9) | <0.001 |
| Diet intake, n (%) | <500 kcal/d | 1,629 (5.0) | 0 (0) | <0.001 |
|  | 500-1000 kcal/d | 1,295 (4.0) | 0 (0) |  |
|  | ≥1000 kcal/d | 29,454 (91.0) | 3,150 (100) |  |

**Notes**:

1. **Abbreviation**: **BMI**, body mass index; **FBG**, fasting blood glucose; **TC**, total cholesterol**; TG**, Triglycerides; **ALT**, alanine transferase; **AST**, aspartate transaminase; **AKP**, alkaline phosphatase; **GGT**, gamma glutamyl-transferase; **TBIL**, total bilirubin; **DBIL,** direct bilirubin; **eGFR-EPI**, estimated glomerular filtration rate calculated by Chronic Kidney Disease Epidemiology Collaboration equation; **CRP**, C reactive protein; **WBC**, white blood cell count; **CCI**, Charlson Comorbidity index.

2. CCI was determined without information on HIV infection or AIDS.

3. If continuous data was in abnormal distribution, data was shown as median and quartile range. Categorical data was shown as number and proportion.

**Supplemental Table 2.** The primary diseases for hospital admission

| Category | Number of participants |
| --- | --- |
| Neurological diseases | 5,549 |
| Gastrointestinal diseases | 18,497 |
| Cardiovascular diseases | 1,738 |
| Pulmonary diseases | 796 |
| Endocrinological diseases | 942 |
| Bone and Joint Disease | 282 |
| Reproductive system disease | 81 |
| Hematological diseases | 3,402 |
| Renal diseases | 1,026 |
| Liver diseases | 339 |
| Malnutrition and electrocyte disorders | 2,426 |
| Others | 447 |

**Supplemental Table 3**. The association between vitamin D and inflammatory biomarkers in 35,528 Chinese adult hospitalized patients: stratified by sex and age

| Inflammatory biomarkers | Group | Serum level of 25-(OH)-D (ng/ml) | | | Each SD increment  (≈7.4 ng/ml) | *P _trend_* |
| --- | --- | --- | --- | --- | --- | --- |
|  |  | <12 | 12-20 | ≥20 |  |  |
| WBC | Men | 0.31 (0.09, 0.53) | 0.05 (-0.13, 0.23) | **Ref (0)** | -0.13 (-0.22, -0.05) | 0.001 |
|  | Women | 0.32 (-0.0001, 0.65) | 0.03 (-0.26, 0.32) | **Ref (0)** | -0.13 (-0.26, -0.004) | 0.044 |
|  | <65 y | 0.57 (0.32, 0.82) | 0.11 (-0.1, 0.31) | **Ref (0)** | -0.24 (-0.34, -0.14) | <0.001 |
|  | ≥65 y | -0.14 (-0.42, 0.12) | -0.01 (-0.33, 0.14) | **Ref (0)** | 0.04 (-0.06, 0.14) | 0.45 |
|  | Spring | 0.27 (-0.17, 0.71) | 0.16 (-0.25, 0.58) | **Ref (0)** | -0.13 (-0.31, 0.04) | 0.14 |
|  | Summer | 0.49 (0.11, 0.86) | 0.04 (-0.26, 0.34) | **Ref (0)** | -0.19 (-0.33, -0.05) | 0.008 |
|  | Autumn | 0.01 (-0.23, 0.25) | -0.09 (-0.28, 0.10) | **Ref (0)** | -0.003 (-0.09, 0.09) | 0.94 |
|  | Winter | 0.60 (0.11, 1.08) | 0.15 (-0.31, 0.62) | **Ref (0)** | -0.25 (-0.44, -0.05) | 0.01 |
| CRP | Men | 1.9 (0.99, 2.82) | 1.41 (0.65, 2.17) | **Ref (0)** | -0.69 (-1.03, -0.35) | <0.001 |
|  | Women | 1.1 (0.13, 2.04) | 0.43 (-0.43, 1.28) | **Ref (0)** | -0.50 (-0.88, -0.12) | 0.009 |
|  | <65 y | 1.43 (0.62, 2.24) | 1.15 (0.47, 1.84) | **Ref (0)** | -0.54 (-0.85, -0.22) | <0.001 |
|  | ≥65 y | 1.22 (0.07, 2.37) | 0.56 (-0.44, 1.57) | **Ref (0)** | -0.54 (-0.97, -0.12) | 0.01 |
|  | Spring | 2.37 (0.83, 3.91) | 0.62 (-0.84, 2.08) | **Ref (0)** | -0.84 (-1.45, -0.23) | 0.006 |
|  | Summer | 1.17 (-0.03, 2.38) | 1.33 (0.37, 2.29) | **Ref (0)** | -0.64 (-1.09, -0.19) | 0.005 |
|  | Autumn | 1.28 (0.09, 2.45) | 0.95 (0.02, 1.88) | **Ref (0)** | -0.39 (-0.82, 0.04) | 0.07 |
|  | Winter | 1.01 (-0.64, 2.66) | 1.23 (-0.34, 2.82) | **Ref (0)** | -0.58 (-1.24, 0.08) | 0.08 |

**Note**:

1. Multivariate model was adjusted by sex (men ***vs.*** women), age (year), hospital (Ren Ji ***vs.*** Xin Hua), season (spring, summer, autumn, or winter), BMI (<18.5 kg/m^2^, 18.5-23.9 kg/m^2^, or ≥24.0 kg/m^2^), anemia (yes ***vs.*** no), albumin (<35.0 g/L ***vs.*** ≥35.0 g/L), fasting blood glucose (<6.0 mmol/L ***vs.*** ≥6.0 mmol/L), serum level of liver enzymes (normal ***vs.*** abnormal), cholestasis (yes ***vs.*** no), eGFR-EPI (<60.0 ml/min/1.73m^2^ ***vs.*** ≥60.0 ml/min/1.73m^2^), dyslipidemia (yes ***vs.*** no), CCI (0, 1-2, or ≥3 points), diet intake (<500 kcal/d, 500-1000 kcal/d, or ≥1000 kcal/d). However, sex was not adjusted when the analysis was stratified by sex.

2. Anemia was confirmed as following: serum hemoglobin<120 g/L in men, or <110 g/L in women. Abnormal liver enzyme was confirmed if any of the following: alanine transferase≥120 U/L, or aspartate transaminase≥150 U/L, or alkaline phosphatase≥250 U/L, or gamma glutamyl-transferase≥100 IU/L. Cholestasis was confirmed if either of the following: total bilirubin≥34.2μmol/L or direct bilirubin≥13.6 μmol/L. Dyslipidemia was confirmed if either of the following: total cholesterol≥5.72 mmol/L or total triglycerides≥1.7 mmol/L.

**Supplemental Table 4**. The association between vitamin D and inflammatory biomarkers in Chinese adult hospitalized patients: sensitivity analysis

| Inflammatory biomarkers | Serum level of 25-(OH)-D (ng/ml) | | | Each unit increment | *P _trend_* |
| --- | --- | --- | --- | --- | --- |
| Sensitivity I | <12 (n=7,231) | 12-20 (n=12,236) | ≥20 (n=9,675) |  |  |
| WBC | 0.36 (0.17, 0.55) | 0.09 (-0.07, 0.25) | **Ref (0)** | -0.16 (-0.23, -0.08) | <0.001 |
| CRP | 1.86 (1.19, 2.53) | 1.08 (0.52, 1.64) | **Ref (0)** | -0.78 (-1.04, -0.52) | <0.001 |
| Sensitivity II |  |  |  |  |  |
| WBC | 0.26 (0.07, 0.45) | 0.04 (-0.11, 0.20) | **Ref (0)** | -0.12 (-0.19, -0.04) | 0.002 |
| CRP | 1.31 (0.65, 1.98) | 1.00 (0.43, 1.57) | **Ref (0)** | -0.52 (-0.77, -0.26) | <0.001 |
| Sensitivity III | <12 (n=9,205) | 12-20 (n=13,001) | ≥20 (n=10,172) |  |  |
| WBC | 0.27 (0.07, 0.47) | 0.01 (-0.16, 0.18) | **Ref (0)** | -0.12 (-0.20, -0.04) | 0.002 |
| CRP | 1.41 (0.73, 2.09) | 1.03 (0.44, 1.61) | **Ref (0)** | -0.58 (-0.84, -0.32) | <0.001 |

**Note**:

1. **Sensitivity I**: excluding those whose eGFR-EPI <60 ml/min/1.73m^2^ (n=6,386).

2. **Sensitivity II**: excluding those whose daily calorie intake<1000 kcal (n=2,924).

3. **Sensitivity III**: excluding those recruited from Xin Hua Hospital (n=3,150).

4. Multivariate model was adjusted by sex (men ***vs.*** women), age (year), hospital (Ren Ji ***vs.*** Xin Hua), season (spring, summer, autumn, or winter), BMI (<18.5 kg/m^2^, 18.5-23.9 kg/m^2^, or ≥24.0 kg/m^2^), anemia (yes ***vs.*** no), albumin (<35.0 g/L ***vs.*** ≥35.0 g/L), fasting blood glucose (<6.0 mmol/L ***vs.*** ≥6.0 mmol/L), serum level of liver enzymes (normal ***vs.*** abnormal), cholestasis (yes ***vs.*** no), eGFR-EPI (<60.0 ml/min/1.73m^2^ ***vs.*** ≥60.0 ml/min/1.73m^2^), dyslipidemia (yes ***vs.*** no), CCI (0, 1-2, or ≥3 points), diet intake (<500 kcal/d, 500-1000 kcal/d, or ≥1000 kcal/d).

5. Anemia was confirmed as following: serum hemoglobin<120 g/L in men, or <110 g/L in women. Abnormal liver enzyme was confirmed if any of the following: alanine transferase≥120 U/L, or aspartate transaminase≥150 U/L, or alkaline phosphatase≥250 U/L, or gamma glutamyl-transferase≥100 IU/L. Cholestasis was confirmed if either of the following: total bilirubin≥34.2 μmol/L or direct bilirubin≥13.6 μmol/L. Dyslipidemia was confirmed if either of the following: total cholesterol≥5.72 mmol/L or total triglycerides≥1.7 mmol/L.
